# Origin of Serum Affects Quality of Engineered Tissues Produced by the Self-Assembly Approach

**DOI:** 10.1155/2016/3825645

**Published:** 2016-05-16

**Authors:** Stéphane Chabaud, Melissa Simard, Isabelle Gendreau, Roxane Pouliot, Stéphane Bolduc

**Affiliations:** ^1^Centre LOEX de l'Université Laval, Génie Tissulaire et Régénération, Centre de Recherche FRQS du CHU de Québec, Axe Médecine Régénératrice, Quebec City, QC, Canada G1J 1Z4; ^2^Faculty of Pharmacy, Laval University, Quebec City, QC, Canada G1J 1Z4; ^3^Department of Surgery, Faculty of Medicine, Laval University, Quebec City, QC, Canada G1J 1Z4

## Abstract

Despite the emergence of serum-free media for cell culture, the use of serum to supplement the culture media is still essential in order to produce engineered urologic tissues using the self-assembly approach, not only for the stromal compartment but also for the uroepithelium. Effects of sera on thickness of these two compartments were measured and quality of the epithelial differentiation was evaluated. For bladder mucosa substitute reconstruction, the use of postnatal sera failed to produce an adequate uroepithelium whereas the fetal sera supplementation did. Postnatal sera also provided thinner stromal compartments than the one obtained using fetal sera, no matter if the fibroblasts from healthy or psoriatic donors were used to reconstruct human skin substitutes.

## 1. Introduction

The balance between the increasing demand of organ transplantation and the availability of such organs is, for now, playing against the patients. On the other hand, too many studies failed to translate from bench to patients because of inaccurate research models: too simplistic, as two-dimensional cell culture, or too complex or physiologically different, as animal models. These problems could be, at least partially, solved by tissue engineering (TE). In fact, the aims of TE are, first, to provide alternative and safer sources of graftable tissues in a context of organ low availability compared to the demand and, second, to set up more accurate tridimensional human models for the fundamental research [1, 2].

In contrast to the vast majority of TE techniques, which use synthetic or biologic scaffolds seeded or not with cells, the self-assembly approach allows the production of tissues using cells from the patient without the need of any exogenous material, ruling out most of safety issues raised by the regulatory agencies. In the presence of ascorbate, mesenchymal cells from dermis [3, 4], cornea [5], bladder [6], adipose tissue [7, 8], and Wharton's jelly of umbilical cord [9] are able to produce, secrete, and assemble their own extracellular matrix (ECM). The self-assembly approach allows the reconstruction of various tissues such as the three layers of skin [7, 10], blood vessels [11], bladders [4, 6], urethras [12, 13], or corneas [14]. Moreover, when placed in a near physiological environment, the mesenchymal, endothelial, or epithelial cells present in these engineered tissues could mimic several pathologies such as fibrotic diseases (wound healing and scars [15], scleroderma [16]), inflammatory diseases (psoriasis [17, 18]), cancers (melanoma [19]), or degenerative disease (amyotrophic lateral sclerosis [20], Fuchs endothelial corneal distrophy [21]).

Culture media for TE are supplemented with sera from various origins, especially fetal calf serum (FCS), newborn calf serum (NCS), and bovine growth serum (BGS). In the self-assembly approach, two steps are affected by the serum used: ECM deposition to produce the stromal sheets as well as epithelium growth and differentiation. Studies have examined the effects of the origin of the mesenchymal [6, 22] and epithelial cells [23] on the quality of tissues produced by the self-assembly technique but not the effects of the origin of the serum. The aim of this study was to determine if the serum origin had a significant impact on the quality of bladder and skin substitutes produced by the self-assembly technique.

## 2. Materials and Methods

### 2.1. Human Cell Culture

All procedures involving patients were conducted according to the Helsinki declaration and were approved by the local Research Ethics Committee. Informed consent of donors was obtained for each specimen. Dermal fibroblasts (Fb) were used as mesenchymal cells to produce the stroma [4]. Skin biopsies were collected from healthy or psoriasis-affected donors. The skin specimen was washed in phosphate buffered saline (PBS) containing 100 U/mL penicillin (Sigma, Oakville, Canada), 25 mg/mL gentamicin (Schering, Pointe-Claire, Canada), and 0.5 mg/mL fungizone (Bristol-Myers Squibb, Montreal, Canada), and then it was cut in small pieces of 1 mm. It was incubated at 4°C overnight with 10 mL of a solution containing 500 mg/mL thermolysin (Sigma) in HEPES buffer with 1 mM CaCl_2_, pH 7.4. The next day, the dermis was manually separated from the epidermis and incubated for 3 hours at 37°C with agitation in a solution of 0.125 U/mL collagenase H (Roche Diagnostics Canada, Montreal, Canada) diluted in Dulbecco-Vogt modification of Eagle's medium (DMEM, Invitrogen, Burlington, Canada) containing 10% fetal bovine serum (Hyclone, Logan, UT), 100 U/mL penicillin, and 25 mg/mL gentamicin (Fb medium). Fb collected by centrifugation were seeded in culture flask at 6 × 10^4^ cells/cm^2^ in Fb medium and cultivated at 37°C in a humidified 8% CO_2_ atmosphere. Medium was exchanged three times a week.

For epithelial cells [4], urothelial cells (UCs) were extracted from a human bladder biopsy and keratinocytes (KCs) from skin biopsies of healthy or psoriasis-affected donors. Until separation of stroma and epithelium, the same process was followed for both epithelia. Epithelium was incubated for 30 min in trypsin (Intergen Company, Purchase, NY, USA). After epithelium dissociation, UCs or KCs were seeded at a density of 5 × 10^5^ cells in 75 cm^2^ culture flasks with 1.5 × 10^5^ irradiated murine Fb (Suis 3T3) as a feeding layer and cultivated in DMEM-Ham containing 5 mg/mL insulin (Sigma), 0.4 mg/mL hydrocortisone (Calbiochem, San Diego, CA, USA), 10^−10 ^M cholera toxin (Sigma), 10 mg/mL epidermal growth factor (Austral Biologicals, San Ramon, CA, USA), 100 U/mL penicillin, and 25 mg/mL gentamicin (Basal medium). For UCs, this basal medium was supplemented with 10% fetal bovine serum (Hyclone, Logan, UT) (UC medium). For KCs, the basal medium was supplemented with 5% Fetal Clone II serum (Hyclone, Scarborough, Ontario, Canada) (KC medium). Cells were placed in a 37°C humidified incubator containing 8% CO_2_. The medium was changed three times a week.

### 2.2. Rabbit Cell Culture

All procedures involving animals were approved by the local Research Ethics Committee. Fibroblasts from rabbit dermis were isolated using the same protocol as in Section 2.1 except incubation and culture were maintained at 38.5°C, their physiologic body temperature [24]. Epithelial cells were not isolated for these experiments.

### 2.3. Stroma Reconstruction

Cultured Fb at passage three were seeded at confluence (4 × 10^5^ Fb) in 6-well plates including a paper anchorage device and cultured in the Fb medium supplemented with 50 *μ*g/mL ascorbate for 28 days to form ECM sheets. For these experiments 8 serum lots were assayed (Table 1). Three Fb sheets were superimposed and cultured for four additional days to allow sheets fusion.

### 2.4. Human Substitutes Production

#### 2.4.1. Human Bladder Mucosa Substitute (BMS) Production

**Figure 1:**
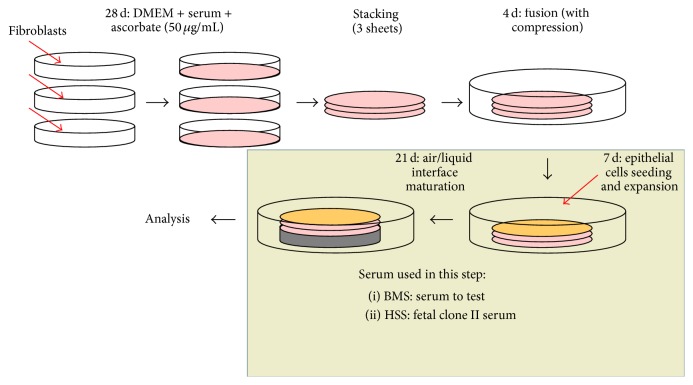
Experimental schemas of bladder mucosa substitute production by the self-assembly approach. The self-assembly technique to produce tissues could be divided into two phases. The first is the production of stroma (32 days, upper part of schema). The second is the proliferation (horizontal expansion) and differentiation (vertical expansion) of the uroepithelium (28 days, lower part of the schema).

Cultured UCs at passage 3 were seeded on top of Fb constructs at a concentration of 3 × 10^4^ cells/cm^2^. The seeded equivalents were cultured in UC medium (we used the same serum lot used for stroma reconstruction) supplemented with 50 *μ*g/mL ascorbate for 7 days under submerged conditions to allow horizontal expansion of UC and full coverage of the stromal constructs. BMS were then elevated at the air-liquid interface for 21 days. UC medium containing ascorbate was used for the air-liquid culture phase (Figure 1). All cultures were done in a 37°C humidified incubator containing 8% CO_2_ and medium was exchanged three times a week.

#### 2.4.2. Human Skin Substitute (HSS) Reconstruction Using Cells from Healthy or Psoriatic Donors

HSS reconstruction was done as previously described [17]. For these experiments, KC medium was used instead of UC medium. The important point to remember is that, in this kind of reconstruction, the epithelium differentiation takes place in the presence of 5% Fetal Clone II serum and not in the presence of tested sera. Only the stroma was assembled with tested sera. Except for this point, cultures followed the same process as for BMS.

### 2.5. Rabbit Stromal Sheet Production

It is the same as in Section 2.3 but the incubator temperature was set at 38.5°C.

### 2.6. Histological Analysis

Sections of each sample were fixed in Histochoice Tissue Fixative (Amresco, Solon, OH) and embedded in paraffin. Histological sections of 5 *μ*m were cut and stained using Masson's Trichrome (MT) or Hematoxylin Eosin (HE).

### 2.7. Thickness Determination

Thicknesses of stroma and epithelium were assessed using pictures taken with Axio Imager M2 microscope (Carl Zeiss) and analyzed by ImageJ software (NIH, Bethesda, MD). 15 measurements were performed per picture. Four tissues (1 representative picture each) were evaluated for BMS, 3 tissues for HSS (using 2 healthy and 2 psoriatic sets of cells, 1 representative picture each), or 3 tissues for rabbit stromas (2 representative pictures each).

### 2.8. Statistical Analysis

Values were expressed as mean ± standard error of the mean. Statistical analysis was conducted using Student's *t*-test to compare two data sets. The level of significance was established at *p* < 0.05.

## 3. Results

### 3.1. Fetal or Newborn Calf Sera Supplementation Increased ECM Deposition Compared to Bovine Growth Sera

**Figure 2:**
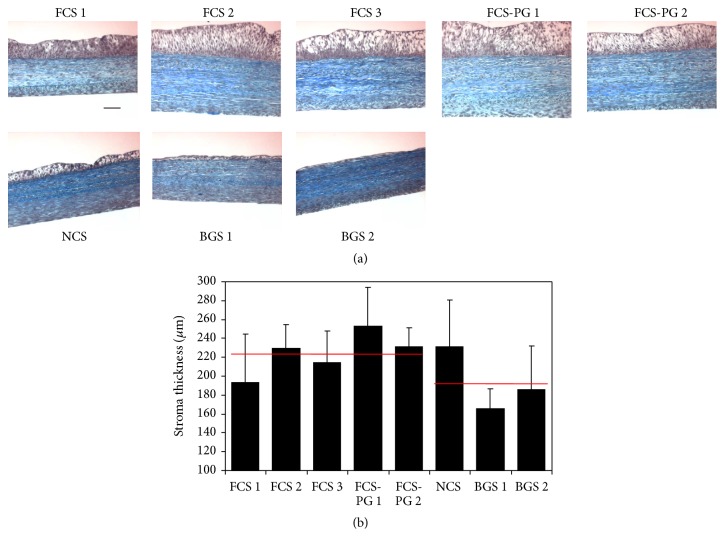
FCS and NCS provided similar stromal thickness whereas BGS resulted in thinner tissues. (a) Masson's trichrome staining of tissues produced using human Fb and UC. Scale bar = 50 *μ*m. (b) Stromal thickness measurement using ImageJ software (*N* = 4 tissues, *n* = 15 measurements). Red lines represent the average value of stromal thickness measured from tissues reconstructed using fetal (left, FCS) or postnatal (right, NCS and BGS) sera. No statistical difference was found.

After tissue production with various sera (FCS, NCS, and BGS), stromal thickness was assessed on Masson's trichrome stained slices (Figure 2(a)). Four among 5 FCS and the assayed NCS provided thicker tissues than the ones produced using BGS: thickness ranged from 193 to 229 *μ*m for FCS, from 231 to 253 *μ*m for FCS-PG (premium grade), 232 *μ*m for NCS, and from 166 to 186 *μ*m for BGS (Figure 2(b)). Nevertheless, the difference between the stroma's thickness of reconstructed tissues using fetal or postnatal sera was not significant: 224 *μ*m ± 22, *N* = 5 fetal sera versus 194 *μ*m ± 34, *N* = 3 postnatal sera, *p* = 0.27. If NCS was excluded and fetal sera compared only to the adult sera, stromas produced using fetal sera were significantly thicker than the one produced using bovine adult sera: 224 *μ*m ± 22, *N* = 5 fetal sera versus 176 *μ*m ± 14, *N* = 2 adult sera, *p* = 0.04.

### 3.2. Fetal Calf Sera Supplementation Increased Uroepithelial Thickness and Quality Compared to Newborn Calf or Bovine Growth Sera

**Figure 3:**
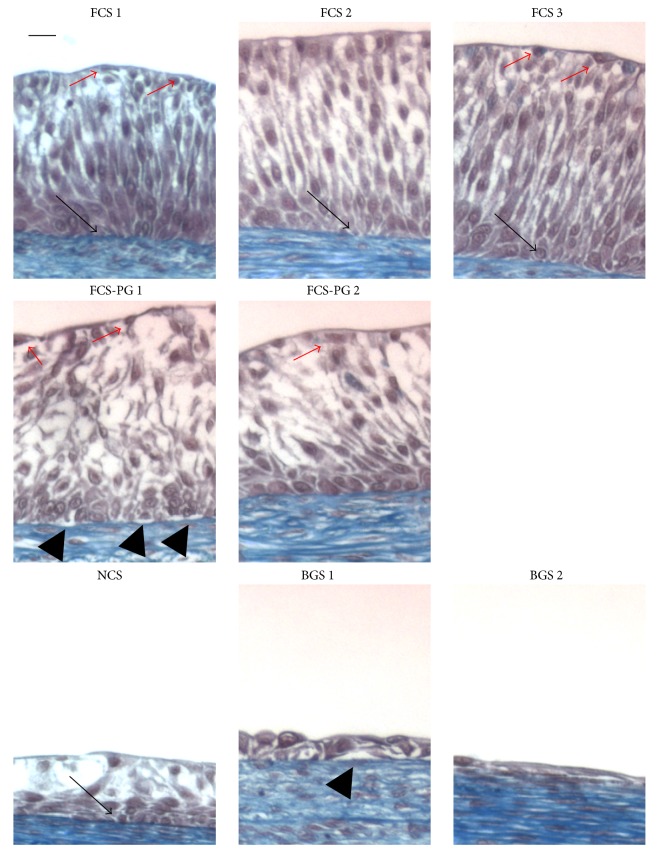
Uroepithelium differentiation was better when FCS was used for cell culture compared to the use of postnatal sera. Masson's trichrome staining of tissues produced using human Fb and UC. Scale bar = 10 *μ*m. Black arrows point to basal lamina (dark blue line after Masson's trichrome staining). Red arrows point clear superficial cells (umbrella cells). Black arrowheads point to detachment of the epithelium from the basal lamina.

**Figure 4:**
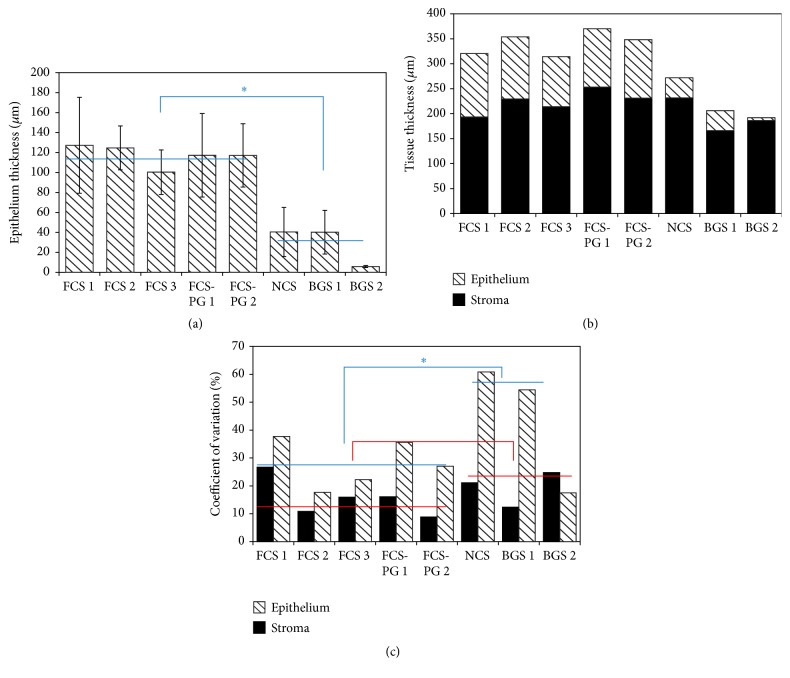
Use of postnatal sera failed to adequately differentiate the uroepithelium. (a) Epithelial thickness measurement using ImageJ software (*N* = 4 tissues, *n* = 15 measurements). Blue lines represent the average value of uroepithelial thickness measured from tissues reconstructed using fetal (left, FCS) or postnatal (right, NCS and BGS) sera. Asterisk is for *p* < 0.05. (b) From Figures 2(b) and 4(a), total thickness of tissue was determined. Part of stroma is in black bars and part of epithelium is in hatched bars. (c) Coefficients of variation of the stromal (black bars) and epithelial (hatched bars) thickness measurements. Blue lines represent the average value of the coefficient of variation of the uroepithelial thickness from tissues reconstructed using fetal (left, FCS) or postnatal (right, NCS and BGS) sera. Red lines are the same for coefficient of variation of stromal thickness. Asterisk is for *p* < 0.05.

The same tissues presented in Figure 2(a) were used to measure the thickness of the uroepithelium. Whereas FCS or FCS-PG gave relatively thick epithelium, NCS and BGS failed to produce a pseudostratified urothelium. The quality of epithelium and the number of layers were adequate for the FCS sera which allowed epithelial proliferation and differentiation (Figure 3): 6 to 8 layers of UCs, with the presence of a dark blue line under the epithelium (basal lamina, black arrows) and flat cells on the top (umbrella cells, red arrows); 6 to 9 layers of UCs obtained with FCS-PG (whereas basal lamina seems lighter and sometimes not well attached to epithelium; e.g.: FCS-PG1; black arrowheads); but 3 to 4 UC layers for NCS and even 1 to 3 UC layers for BGS with no clear basal lamina. Thickness of epithelium ranged from 100 to 127 *μ*m for FCS or FCS-PG but only 40 *μ*m for NCS and from 6 to 40 *μ*m for BGS (Figure 4(a)). Relative portions of epithelial and stromal compartment in the reconstructed tissues were similar between conditions using FCS or FCS-PG but the epithelial layer was significantly reduced in tissues cultivated with NCS or BGS (Figure 4(b)): 117 *μ*m ± 10, *N* = 5 fetal sera versus 29 *μ*m ± 20, *N* = 3 postnatal sera, *p* < 0.01.

### 3.3. Postnatal Sera Resulted in Higher Variability in Epithelial Thickness Than the Fetal Calf Sera

When coefficients of variation of stromal thickness were calculated (Figure 4(c)), they ranged from 9 to 17% for the stroma when tissues were produced using FCS or FCS-PG (except FCS 1, 27%) whereas they ranged from 21 to 25% for the stroma obtained from NCS and BGS supplementation (except for BGS 1, 12%). Difference between the coefficient of variation of the stromal thickness of the tissue produced using fetal or postnatal sera was not significant: 28% ± 8, *N* = 5 fetal sera versus 44% ± 23, *N* = 3 postnatal sera, *p* = 0.35. Coefficients of variation of epithelial thickness showed a statistical significant difference between fetal sera and postnatal sera, which were greater. They ranged from 18 to 38% for the stroma when tissues were produced using FCS or FCS-PG whereas they ranged from 54 to 61% for the stroma resulting from NCS and BGS supplementation (except for BGS 2, 18% but there was virtually no epithelium in this condition). Difference in the coefficient of variation of the epithelial thickness between tissues reconstructed with fetal and postnatal sera was significant (when BGS 2, the one virtually without epithelium, was excluded): 28% ± 9, *N* = 5 fetal sera versus 58% ± 1, *N* = 2 postnatal sera, *p* < 0.001.

### 3.4. Fetal Calf Sera Supplementation Increased Stromal Thickness Compared to Bovine Growth Sera in a Healthy or Psoriatic HSS

**Figure 5:**
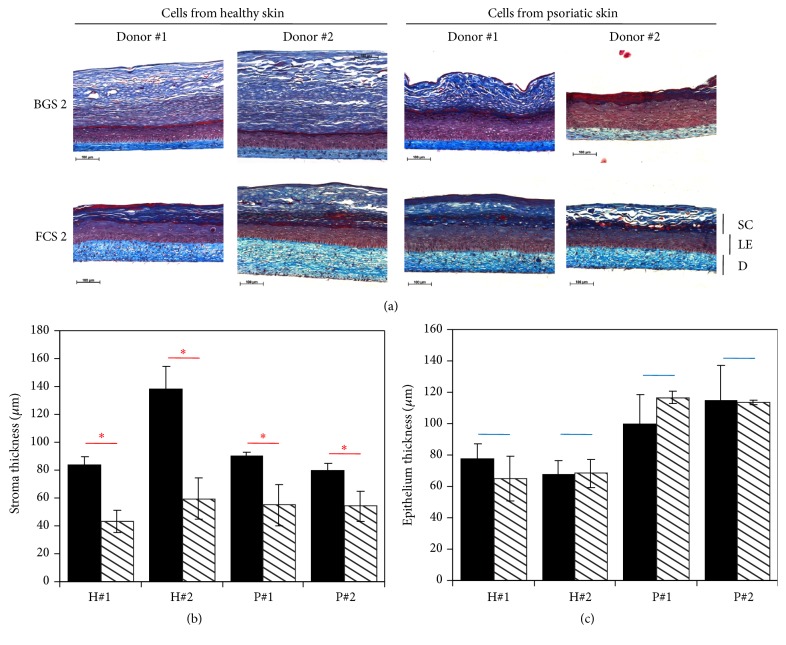
FCS gave thicker engineered stroma from healthy and psoriatic fibroblasts than BGS. (a) Masson's trichrome staining of tissues produced using human Fb and KC. D is for dermis (stromal) part, LE for living epidermis (epithelium), and SC for stratum corneum. Scale bars are as indicated. In (b) and (c), black bars are for skin substitutes reconstructed using FCS2 serum and hatched bars are for skin substitutes reconstructed using BGS2. H#1 and #2 are for tissues reconstructed using fibroblasts and keratinocytes from healthy donors 1 and 2. P#1 and #2 are for tissues reconstructed using fibroblasts and keratinocytes from donors suffering of psoriasis. (b) Stromal thickness measurement using ImageJ software (*N* = 2 donors, *n* = 3 tissues, and *n* = 15 measurements). Red lines and asterisks are for comparison between stromal thickness values of tissues reconstructed with FCS2 or BGS2. Asterisks indicate statistically significant difference (i.e., *p* ≤ 0.05). (c) Epithelial thickness measurement using ImageJ software (*N* = 2 donors, *n* = 3 tissues, and *n* = 15 measurements). Blue lines are for comparison between epithelial thickness values of tissues reconstructed with FCS2 or BGS2.

In this experiment, tissues were reconstructed using two different sera at each step, stroma production and epithelial differentiation. The fetal (FCS2) and postnatal (BGS2) sera were only used in the first part (stroma reconstruction). Cells (Fb and KCs) came from healthy and psoriatic donors (2 for each condition). After tissue production, slices of tissues were stained using Masson's trichrome technique (Figure 5(a)). No matter the origin of cells, healthy or psoriatic, the stromal thickness was increased when FCS was used compared to BGS: 111 *μ*m ± 32, *N* = 2, *n* = 3 fetal sera versus 51 *μ*m ± 15, *N* = 2, *n* = 3 postnatal sera, *p* = 0.01 for tissues reconstructed with healthy cells; 84 *μ*m ± 7, *N* = 2, *n* = 3 fetal sera versus 54 *μ*m ± 12, *N* = 2, *n* = 3 postnatal sera, *p* = 0.002 for tissues reconstructed with psoriatic cells (Figure 5(b), Table 2). No significant difference was observed between the epithelium's thicknesses, no matter if the stroma was reconstructed using fetal or postnatal sera: 73 *μ*m ± 9, *N* = 2, *n* = 3 fetal sera versus 67 *μ*m ± 11, *N* = 2, *n* = 3 postnatal sera, *p* = 0.34 for tissues reconstructed with healthy cells; 109 *μ*m ± 20, *N* = 2, *n* = 3 fetal sera versus 115 *μ*m ± 3, *N* = 2, *n* = 3 postnatal sera, *p* = 0.51 (Figure 5(c), Table 2). As expected, the thickness of living epidermis was 1.5-fold (for FCS) to 1.8-fold (for BGS) thicker when cells from psoriatic donors were used (*p* = 0.01 for FCS and *p* < 0.001 for BGS) (Table 2). Coefficient of variation for stromal thickness was similar for the tissues reconstructed using cells from healthy donors (29%) whereas it was inferior with FCS for tissues reconstructed using cells from psoriatic donors (8% for FCS versus 22% for BGS).

### 3.5. BGS Provided Thinner Reconstructed Rabbit Stroma Than NCS and FCS or FCS-PG but Postnatal Sera Resulted in Greater Variability

**Figure 6:**
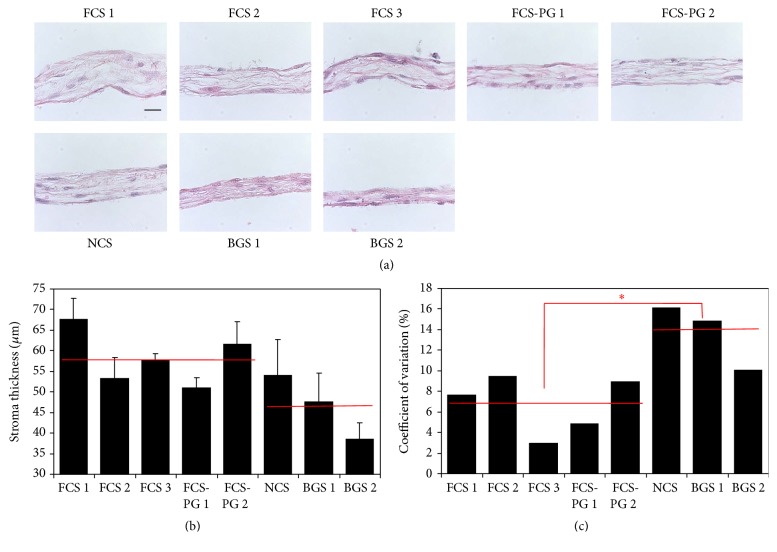
Postnatal sera supplementation resulted in thinner reconstructed rabbit stroma with higher variability than fetal sera use. (a) Hematoxylin and Eosin staining of tissues produced using human Fb. Scale bar = 25 *μ*m. (b) Stromal thickness measurement using ImageJ software (*N* = 3 tissues, *n* = 30 measurements). Red lines represent the average value of stromal thickness measured from tissues reconstructed using fetal (left, FCS) or postnatal (right, NCS and BGS) sera. No statistical difference was found. (c) Coefficients of variation of the stromal thickness measurements. Red lines represent the average value of the coefficient of variation of the stromal thickness from tissues reconstructed using fetal (left, FCS) or postnatal (right, NCS and BGS) sera. Asterisk is for *p* < 0.05.

After tissue production with various sera (FCS, NCS, and BGS), thickness of reconstructed rabbit stroma was assessed on Hematoxylin and Eosin stained slices (Figure 6(a)). Except for BGS, all tissues showed roughly the same thickness ranging from 53 to 67 *μ*m whereas Fb cultivated in the presence of BGS ranged from 38 to 47 *μ*m (Figure 6(b)). Difference between the thickness of stromas reconstructed with rabbit Fb using fetal or postnatal sera was not significant: 58 *μ*m ± 7, *N* = 5 fetal sera versus 47 *μ*m ± 8, *N* = 3 postnatal sera, *p* = 0.1. If NCS was excluded, similarly to the results obtained with human Fb, postnatal sera (NCS and BGS) presented greater coefficients of variation of stromal thickness: 3 to 9% for fetal sera versus 10 to 16% for postnatal ones (Figure 6(c)). Once again, difference between the coefficient of variation of stromal thickness of tissues reconstructed using fetal and postnatal sera was significant: 7% ± 3, *N* = 5 fetal sera versus 14% ± 3, *N* = 3 postnatal sera, *p* = 0.03.

## 4. Discussion

The serum is still an essential component of cell culture to produce engineered tissues using the self-assembly approach because the cells have to produce, deposit, and assemble their own ECM scaffold. The choice of sera is a crucial step in order to obtain stromas of an acceptable quality (i.e., sufficiently thick to be used for grafting or subsequent experiments). Even if it could be omitted for the last steps of epidermis differentiation [25], it remains a key of horizontal and vertical uroepithelial expansion. No previous study on impact of sera origin has been published for the self-assembly technique. Comparison of sera from different origins has been investigated on exogenous bovine collagen lattices gels [26]. Nevertheless, the interesting results obtained could be translated to our model: postnatal sera showed a lower mitogenic potential, therefore a reduced growth rate, and less MMP were produced but collagen synthesis was unchanged. Our previous studies have demonstrated that collagen synthesis was not a good marker of the final thickness of the reconstructed stroma [4] but that MMP could enhance the matrix deposition and could improve the distribution of the cells throughout the tissue [27]. Moreover, the Fb cultivated in postnatal sera were more prone to develop a myofibroblastic phenotype.

The present study showed that the thickness of the stroma produced with human Fb to reconstruct a BMS was very similar whatever the serum used (Figure 2(b)). If only adult sera (BGS) were considered, the thickness of these tissues was inferior when compared to fetal sera. More importantly, uroepithelium could not differentiate when the cell culture was supplemented with postnatal sera (Figures 3 and 4(b)). This inability to promote differentiation of UC in the presence of postnatal sera could result from 2 different main causes. First, the direct influence of the sera: some essential factors for the differentiation of UC could be missing in postnatal sera or on the contrary, some factors which could block the differentiation might be present. Second, the influence of stroma: the quality of the stroma and/or Fb could be altered when using postnatal sera. This could block the proliferation or cause the inability of UC to differentiate in intermediate and superficial layers of the urothelium (the latter which is essential to the barrier function of the bladder epithelium). One of the problems to discriminate between these different causes is that the stroma is a trap for growth factors and peptides. Some of them could be stored and be later released during the epithelial differentiation. Use of two different sera for the two distinct steps of reconstruction could be investigated. For example, the production of a stroma is performed using a fetal serum, which is favourable to the epithelial differentiation, followed by the epithelial differentiation step realized with a postnatal serum, which usually fails to produce a mature epithelium. If the resulting epithelium is adequately differentiated, is it due to the fetal serum-produced stroma/Fb or a release of trapped fetal serum factors? It is difficult to remove trapped factors from the stroma without altering its structure and then its function.

In a second set of experiments, HSS were reconstructed. After seeding KCs on the stroma, a serum known to allow epidermis differentiation was used for all conditions. Only the stromal part was reconstructed using fetal or postnatal sera. Fetal sera resulted in stromas which were clearly thicker than the ones produced using postnatal sera, no matter if the cells were extracted from a healthy or psoriatic donor (Figure 5(b)). No difference could be seen between epithelia supported by a stroma produced using fetal or postnatal sera. But, as expected, epidermis was well differentiated for healthy skin substitutes and hyperproliferative, then thicker, for psoriatic skin substitutes (Figure 5(c)).

The experiments using rabbit Fb provided similar results to what was obtained for BMS and HSS but the use of postnatal sera resulted in a higher variability of the stromal thickness (Figure 6(c)).

In the context of the translation of the self-assembly technique to clinical or fundamental applications, the reproducibility of the tissue quality is essential. Recent analyses have identified more than 4.000 compounds in sera [28]. Use of such an undefined and variable mix could not be a long-term solution [29] even if it is acceptable in a first step. Serum use also raises the concern of potential contamination with pathogenic factors [30], which could be avoided by extensive tests which can, in turn, quickly lead to prohibitive costs. Use of animal-free components, not only in the culture medium but also during the whole process (including extraction, biobanking, cell culture, e.g., medium and trypsination, and tissue reconstruction) should then be recommended [31]. In the future, it is also possible that ethical considerations relative to the animal welfare could reduce the availability of serum. For all these reasons, replacement of medium supplemented with serum by defined medium is needed to overcome these issues. At the present, there exist several commercial and noncommercial serum-free media but, for most of them, they replace the serum by other animal components such as pituitary gland extracts [32]. If in certain cases the mix is more defined, the main problems remain.

Identification of factors present in the fetal sera and which allow thicker stroma and adequate differentiation of epithelium should be the next step in order to produce a defined medium without variability from lot to lot. Because use of adult sera gave thinner stroma and epithelium than the use of fetal ones, comparison between them for their components could help to determine what factors are dispensable or detrimental, even if it will be a huge amount of work.

## 5. Conclusions

Production of reconstructed human tissues by the self-assembly technique requires factors that are present in fetal sera but not in postnatal sera. Moreover, no matter if the biopsies were taken from a human, healthy or psoriasis-affected, or a rabbit, the obtained stroma was thicker and presented less variation when fetal sera were used compared to adult sera. Fetal serum use remains essential and research to discover what the key factors are will allow production of a more defined medium.

## Figures and Tables

**Table 1:** Sera used in this study. ID is for identity. Origin is the development stage of the calf when serum has been collected.

ID	Origin	Lot	Country of origin	Company
FCS 1	Fetal	15A086	Canada	Sigma
FCS 2	Fetal	115681	USA	Wisent
FCS 3	Fetal	115676	USA	Wisent
FCS-PG 1	Fetal	253B14	USA	Seradigm
FCS-PG 2	Fetal	351B14	USA	Seradigm
NCS	Neonatal	1353143	New Zealand	Life Technologies
BGS 1	Postnatal	AXM54404	USA	Hyclone
BGS 2	Postnatal	AYK170734	USA	Hyclone

**Table 2:** Thickness values for tissue reconstructed with FCS2 and BGS2. BMS is for bladder mucosa substitute and HSS for human skin substitute. (hea) is for tissues reconstructed with stromal and epithelial cells from healthy people; (pso) is for tissues reconstructed with stromal and epithelial cells from lesional sites of the psoriasis-affected people.

Reconstructed tissue compartment	Average thickness, FCS2 (*µ*m)	Average thickness, BGS2 (*µ*m)	Thickness ratio (FCS2/BGS2)	*p* value Student's *t*-test
BMS stroma	229 ± 25	186 ± 46	1.2	0.16
BMS urothelium	124 ± 22	6 ± 1	20.6	0.002
HSS stroma (hea)	111 ± 32	51 ± 15	2.2	0.01
HSS stroma (pso)	84 ± 7	54 ± 12	1.6	0.002
HSS epidermis (hea)	73 ± 9	67 ± 11	1.1	0.34
HSS epidermis (pso)	109 ± 20	115 ± 3	0.9	0.51
Rabbit stroma	53 ± 5	38 ± 4	1.4	0.02
